# Field effectiveness of new visceral leishmaniasis regimens after 1 year following treatment within public health facilities in Bihar, India

**DOI:** 10.1371/journal.pntd.0007726

**Published:** 2019-09-26

**Authors:** Vishal Goyal, Sakib Burza, Krishna Pandey, Shambhu Nath Singh, Ravi Shankar Singh, Nathalie Strub-Wourgaft, Vidya Nand Rabi Das, Caryn Bern, Allen Hightower, Suman Rijal, Temmy Sunyoto, Fabiana Alves, Nines Lima, Pradeep Das, Jorge Alvar

**Affiliations:** 1 Drugs for Neglected Diseases *initiative* (DND*i*), New York, United States of America; 2 Médecins Sans Frontières (MSF), New Delhi, India; 3 Rajendra Memorial Research Institute of Medical Sciences (RMRI), Patna, Bihar, India; 4 Sadar Hospital Chapra, Saran, Bihar, India; 5 Drugs for Neglected Diseases *initiative* (DND*i*), Geneva, Switzerland; 6 University of California San Francisco, San Francisco, California, United States of America; 7 Independent consultant, Bangkok, Thailand; 8 Drugs for Neglected Diseases *initiative* (DND*i*), New Delhi, India; 9 Médecins Sans Frontières (MSF), Spain; Saudi Ministry of Health, SAUDI ARABIA

## Abstract

**Background:**

An earlier open label, prospective, non-randomized, non-comparative, multi-centric study conducted within public health facilities in Bihar, India (CTRI/2012/08/002891) measured the field effectiveness of three new treatment regimens for visceral leishmaniasis (VL): single dose AmBisome (SDA), and combination therapies of AmBisome and miltefosine (AmB+Milt) and miltefosine and paromomycin (Milt+PM) up to 6 months follow-up. The National Vector Borne Disease Control Program (NVBDCP) recommended an extended follow up at 12 months post-treatment of the original study cohort to quantify late relapses.

**Methods:**

The 1,761 patients enrolled in the original study with the three new regimens were contacted and traced between 10 and 36 months following completion of treatment to determine their health status and any occurrence of VL relapse.

**Results:**

Of 1,761 patients enrolled in the original study, 1,368 were traced at the extended follow-up visit: 711 (80.5%), 295 (83.2%) and 362 (71.5%) patients treated with SDA, AmB+Milt and Milt+PM respectively. Of those traced, a total of 75 patients were reported to have relapsed by the extended follow-up; 45 (6.3%) in the SDA, 25 (8.5%) in the AmB+Milt and 5 (1.4%) in the Milt+PM arms. Of the 75 relapse cases, 55 had already been identified in the 6-months follow-up and 20 were identified as new cases of relapse at extended follow-up; 7 in the SDA, 10 in the AmB+Milt and 3 in the Milt+PM arms.

**Conclusion:**

Extending follow-up beyond the standard 6 months identified additional relapses, suggesting that 12-month sentinel follow-up may be useful as a programmatic tool to better identify and quantify relapses. With limited drug options, there remains an urgent need to develop effective new chemical entities (NCEs) for VL.

## Author summary

In 2010, the WHO Expert Committee recommended liposomal amphotericin B (in single or multiple doses) along with three short combination treatment regimens containing liposomal amphotericin B (LAmB), miltefosine (Milt) and/or paromomycin (PM) as preferred options to replace the existing miltefosine monotherapy for kala-azar treatment in South Asia. The Drugs for Neglected Diseases *initiative* (DND*i*) in partnership with Rajendra Memorial Research Institute of Medical Science (RMRI-Regional ICMR institute), State Health Society Bihar, and Médecins Sans Frontières (MSF) conducted a phase 4 field effectiveness study to determine the effectiveness and to assess the safety and feasibility of using single dose LAmB, and the combination therapies of LAmB+Milt and Milt+PM for the treatment of VL at public healthcare facility settings in India. Based on the provisional results of this effectiveness study at 6 months follow-up, the Indian government revised the national policy in August 2014, introducing SDA as first option and Milt+PM as second option to replace miltefosine monotherapy in the kala-azar elimination initiative. National Vector Borne Disease Control Programme (NVBDCP) expert committee recommended to follow up this large cohort of patients for one year to identify relapses yielding further evidence on new treatment regimens.

## Introduction

Leishmaniasis is a disease caused by infection of protozoa parasites *Leishmania*, transmitted through the bite of phlebotomine sand flies. The visceral leishmaniasis form affects the reticuloendothelial system, presenting insidious clinical manifestations of fever, hepato-and splenomegaly, anemia and weight loss. Visceral leishmaniasis is fatal if not treated. In Asia, visceral leishmaniasis, also named kala-azar, affects poor populations mainly in India, Bangladesh and Nepal. Effective treatment is key to improving patient outcomes and reducing disease transmission [1]. Current treatment recommendations in Asia include liposomal Amphotericin B (LAmB), or combination therapies containing Amphotericin B (LAmB), miltefosine (Milt) and/or paromomycin (PM) [1]. Shorter regimens appear to be more affordable, safer, easier to administer and with better compliance, and combinations may protect the lifespan of the individual drugs [2–4]. Based on effectiveness study results [5], the Indian government has adopted single dose LAmB as first option for VL treatment, and Milt+PM as second option to replace miltefosine monotherapy in the kala-azar elimination initiative since 2014.

The majority of phase 3 clinical trials of treatments for VL use 6 months following completion of treatment as the endpoint for efficacy assessment, which has also been adopted by the WHO as the programmatic indicator of final cure rates in routine national programmes. However, studies in India have shown that a longer follow-up period of up to 12 months may identify further cases of relapse [6,7]. The occurrence of Post Kala-azar Dermal Leishmaniasis (PKDL), which is also a VL sequel of interest, is usually observed at an average of 2 years after VL treatment in India; thus, continued follow-up beyond 12 months and a future analysis are planned to ascertain incidence of PKDL [8]. In order to determine the incidence of relapse at 12-months following completion of treatment in these new treatment regimens, the National Vector Borne Disease Control Programme (NVBDCP) expert committee recommended conducting an additional follow up of the cohort of patients from the original study (N = 1,761) at one-year post VL treatment. This manuscript presents the results of this extended follow-up.

## Materials and methods

The original study has been described in a previous publication (5). All the 1,761 patients who were treated in the original non-randomized, non-comparative study with single dose AmBisome (SDA), a combination of AmBisome and miltefosine (AmB+Milt) or a combination of miltefosine and paromomycin (Milt+PM), were contacted and invited to attend one of the original study centres at 12 months post-treatment. Details of inclusion and exclusion criteria, VL treatment regimens, study sites, etc. are described in Goyal et al., 2018 [5].

### Patient follow-up

Patients who had received VL treatment with any of the three treatment regimens were contacted by the Information, Education and Communication (IEC) team by telephone to attend to a 12-months follow-up visit. If a patient did not come for the visit, the IEC team would contact them by telephone again and if unable to make contact, conduct a home visit with the support of ASHA (Accredited Social Health Activists) workers. Follow-up visits were performed between January and September 2015. Follow-up was intended to take place 12 months after completion of treatment. However, due to the long patient recruitment period of the original study (August 2012 to October 2014), by the time of the request by the NVBCP to extend follow- up and given the shorter time frame in which to complete the follow-up work, many of the patients had already passed the 12 month post-treatment time point. As a result, the 12-month follow-up visits were performed between a range of 10 and 36 months post treatment completion (S1 Table).

During the extended follow-up visit, patients were assessed clinically by medical history, physical exam and haemoglobin analysis. Parasitological diagnosis (bone marrow or spleen aspiration) was indicated if VL signs and symptoms were present.

### Ethics statement

The study was approved by the Institutional Ethics Committee of RMRI, Patna. Patients from the original study were contacted by telephone and verbally consented to participate in the study while attending the secondary follow up visit. Those who attended were consented in writing by treating physician and those who agreed were included in the analysis. For children, consent of parents or of a legal representative was obtained.

### Data analysis and statistical methods

Analyses were performed (excluding treatment interruptions / defaults in the original study, PKDL and those lost to follow-up at the extended follow up). Analyses were also stratified by age (≤ 12 and > 12 years).

Effectiveness outcomes were characterized as cured, relapse, death or lost to follow-up (if no contact was made).

Factors associated with VL relapse any time up to the extended follow-up were analysed. These analyses compared only those with extended follow-up data and excluded patients with earlier default, treatment interruption or PKDL.

Analyses were conducted in SAS 9.320 (SAS Institute, Cary, NC, USA). Statistical differences were tested in univariate analyses using Chi Square test, Fisher Exact test, Wilcoxon Rank Sum, or Kruskal-Wallis tests as appropriate. Multivariable logistic regression models were constructed using stepwise backwards variable elimination, and model fit tested using the Hosmer and Lemeshow Goodness-of-Fit Test.

**Fig 1:**
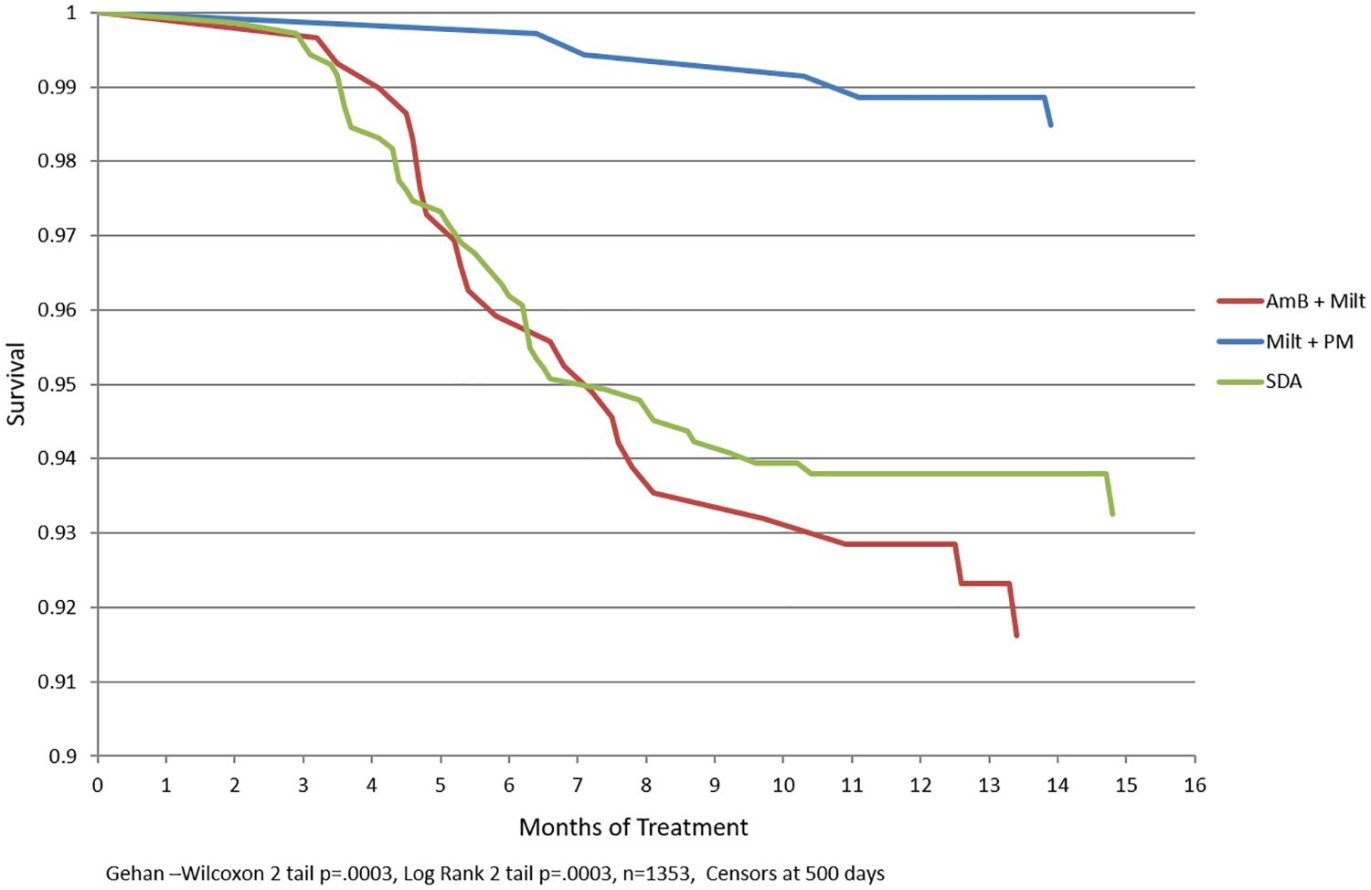
Kaplan Meier curve for 12 month treatment by drug.

Time to relapse was used to compute survival or Kaplan Meier curves by treatment arm. Kaplan Meier curves (Fig 1) were compared using both Wilcoxon Gehan statistics (which emphasize early differences in hazard rates), and the Log Rank test (which is more sensitive to later deviations in hazard rates). Log-log plots of the survival distribution function were produced that indicated the proportional hazards assumption was reasonable. Accordingly, proportional hazards models, using the time until relapse as an outcome, were constructed using stepwise backwards variable elimination.

### Outcome definitions

**Table:** 

*Final cure*	No recurrence of VL symptoms and absence of parasitologically confirmed relapse by extended follow-up visit.
*Relapse*	Recurrence of clinical symptoms and visualization of parasites in spleen or bone marrow aspirate anytime between end of treatment up to extended follow-up visit.
*Defaulter*	Failure to finish treatment against medical advice.
*Lost to follow-up*	Unable to be traced/no contact at the extended follow up visit.

## Results

Baseline characteristics of the population was presented in *Goyal et al*. *2018* [5]. Main findings were severe anaemia was more common in the SDA treatment arm. ALT levels were higher in the AmB+Milt arm, whereas AST levels were higher in the SDA and AmB+Milt arms than in the Milt+PM arm. Patients treated with SDA and Milt+PM (a majority of whom were treated at district hospitals) tended to be younger, more likely to be female, and to present with severe wasting than those treated with AmB+Milt, but these differences did not reach statistical significance. The limited number of patients treated at the RMRIMS had a significantly longer reported duration of illness (median of 8 weeks as compared to 4 weeks in other sites).

A total of 1,368 patients were successfully traced and attended the extended follow-up visit. Of these, 15 patients had developed PKDL, these cases were excluded from subsequent analyses. Accordingly 1353 patients were included in the current analyses: 710 treated with SDA (79.7% of the original 891), 294 treated with AmB+Milt (82.1% of the original 358) and 349 treated with Milt+PM (68.1% of the original 512). Successful tracing at extended follow up was significantly lower in the Milt+PM arm compared to the other two arms. Median time to extended follow-up was 12.6 months (IQR 12.1–14.5) for the SDA group, 13.1 months (IQR 12.3–16.0) for the AmB+Milt group and 10.3 months (IQR 7.1–11.1) for the Milt+PM group.

Of 1353 patients with extended follow-up, 75 reported a history of relapse; 45 (6.3%) of 710 in the SDA arm, 25 (8.5%) of 294 in the AmB+Milt arm and 5 (1.4%) of 349 patients in the Milt+PM arm. Of the 75 relapse cases, 55 had already been identified in the 6-months follow-up and 20 were identified as new cases of relapse at extended follow-up; 7 in the SDA, 10 in the AmB+Milt and 3 in the Milt+PM arms.

Interestingly, at 6 months follow up, cure rate by complete case analysis of SDA was 95.5%, AmB+Milt was 95.5% and Milt+PM 99.6% [Goyal et al, 2018], meanwhile at 12 months, the cure rate decreased in all arms to 93.7%, 91.5% and 98.6%, respectively.

In univariate, only the drug regimen used, age ≤ 12 years, and length of illness less than 8 weeks were significantly associated with risk of relapse (Table 1). Gender, anaemia, wasting, abnormal liver/renal function tests and a previous history of VL were not associated with relapse. S2 Table shows proportion of relapses between 0–6 months, 6–12 months and > 12 months.

**Table 1:** Univariate analyses of factors associated with VL relapse by 12 months N = 1353.

	Relapse by extended follow up (N = 75)	Cured at extended follow up (N = 1,278)	p value
	n (row %)	n (row %)	
**Regimen**			
SDA	45 (6.3)	665 (93.7)	0.0002
Amb+Milt	25 (8.5)	269 (91.5)	
Milt+PM	5 (1.4)	344 (98.6)	
**Sex**			
Male	50 (6.1)	774 (93.9)	0.29
Female	25 (4.7)	504 (95.3)	
**Age**			
2–12 years	34 (7.8)	400 (92.2)	0.01
>12 years	41 (4.5)	878 (95.5)	
**Reported length of illness**			
≤8 weeks	70 (6.5)	1004 (93.5)	0.002
>8 weeks	5 (1.8)	274 (98.2)	
**Severe anaemia**			
Yes	22 (4.7)	451 (95.4)	0.29
No	53 (6.0)	827 (94.0)	
**Severe wasting**			
Yes	15 (7.3)	191 (92.7)	0.24
No	60 (5.2)	1,087 (94.8)	
**ALT ≥ 200 U/L**			
Yes	1 (1.9)	53 (98.2)	0.23
No	74 (5.7)	1225 (94.3)	
**AST ≥ 200 U/L**			
Yes	6 (4.3)	133 (95.7)	0.5
No	69 (5.7)	1,145 (94.3)	
**Creatinine ≥ 1.5 (units)**			
Yes	2 (6.1)	31 (93.9)	0.89
No	73 (5.5)	1,247 (94.5)	
**Patient category**			
Primary kala-azar	69 (5.4)	1,210 (94.6)	0.59
Previously treated kala-azar	4 (8.7)	42 (91.3)	
Transferred	2 (7.1)	26 (92.9)	

In multivariable logistic regression models, these same three variables remained after stepwise elimination (Table 2). A proportional hazards model yielded similar results.

**Table 2:** Logistic regression model of factors associated with VL relapse by extended follow up (analysis).

Outcome = relapse any time during follow-up compared to cure at extended follow up		
LTFU at extended follow up excluded from analysis. (N = 1,353; 75 relapses)	aOR	95% Confidence Limits
**Regimen**		
Amb+Milt	1.44	0.86, 2.41
Milt+PM	0.21	0.08, 0.55
SDA	Referent	
**Age**		
2–12 years	1.91	1.18, 3.09
>12 years	Referent	
**Reported length of illness**		
≤8 weeks	3.34	1.33, 8.42
>8 weeks	Referent	

Kaplan Meier plots showed highly significant differences in relapse rates between study arms for both the Wilcoxon test (emphasizing early differences) and the Log Rank test (giving more weight to later differences) (p<0.0001 in each case), confirming the observations from the multivariate logistic regression analysis.

There are consistent statistically significant differences in follow up / relapse time by study arm for both cured and relapsed patients. The median relapse time is much later for Milt+PM patients, but subjects that were cured in the Milt+PM arm were also followed up for a longer period. In addition, there are significant differences in follow-up time by site. Fortunately, relapse times are shorter than follow-up times for cured patients.

To attempt to address this issue, survival times of over 500 days were recoded to 500 days (essentially right truncation). The resulting Kaplan Meier plots by study arm are much closer to each other, since relapse rates for all three arms were less than 10% at 500 days, which corresponds more closely to the observed cure rates. The differences were still highly significant between arms (p<0.0003 for both Wilcoxon and Log Rank tests). Since the recoding does not change the ranking of survival (that is, relapse-free follow-up) times, the results for the un-recoded and recoded survival times were virtually identical, despite having Kaplan Meier curves that appeared to be quite different. However, given the differences between follow-up time by drug arm, the differences in survival time are difficult to interpret. It may be difficult to interpret whether these differences are due to follow-up procedures or to actual differences in survival by drug arm.

## Discussion

In the original study, 55 relapses were identified by 6 months follow-up; complete case cure rates were 95.5% for SDA (95% CI 93.9–96.8), 95.5% for AmB+Milt (95% CI 92.7–97.5) and 99.6% for Milt+PM (95% CI 98.6–99.9) [5]. The extension of follow-up in this study allowed for the identification of a further 20 cases of relapse. The cure rates at the extended 12 months follow-up was 93.7% for SDA, 91.5% for AmB+Milt and 98.6% for Milt+PM in the complete case analysis. The possibility of late relapses with all drugs or combinations, should be explained to the patients when discharged.

The efficacy of SDA at 6 months in a study conducted in Bangladesh by local doctors was similar (ITT efficacy 97%) [9]. Earlier, a DND*i* phase-3 clinical trial in Bangladesh assessing the safety and efficacy of short course combination regimens in field conditions at *upazila* (subdistrict) level documented excellent efficacy outcomes (≥95%) at 6 months with very good safety profiles [10].

Similarly, an earlier study showed that following the treatment with 20mg/kg AmBisome in 4 divided doses, the relapse rate was 0.3% at 6 months after treatment and 3.7% by 12 months (70% of all relapses), with the mean point of relapse at 9.6 months [7].

Considering the relatively low proportion of relapses at extended follow up seen in this study, the resources required, cost-effectiveness and feasibility of implementation at programme level may need to be considered when recommending additional routine follow-up of all patients of up to 1 year (in addition to follow up at 6-months). However, a recent study in Nepal showed that the failure rate for miltefosine was 10.8% at 6 months rising to 20% by 12 months, with the age survival analysis consistent with the pharmacokinetics of allometric dosing, which has been shown to result in suboptimal levels in children [6, 11]. Without extended follow up, the increasing failure rates of this regimen may not have been detected. In the present study, the extended follow-up period varied from 10 to 36 months, with 90% of the relapses captured by 20 months of follow-up. As such, it may be that 12-month failure rates may provide a signal of reduced effectiveness when monitoring new drugs, however this requires further research.

In India, PKDL cases could be reservoir of the *Leishmania* parasite and may play major role in anthroponotic transmission of VL [12]. Development of resistance to antimonial monotherapy in South Asia has been well described [13]. Apart from a few case descriptions, there is no strong evidence of resistance to miltefosine from clinical isolates in immunocompetent patients [6]. Nevertheless, the efficacy of miltefosine has decreased from 94% to 90% within a decade of use in India [14] leading to a strong recommendation to be used only in combination with other medicines [15]. The fact that late relapses were found in this extensive cohort, even when used in combination, underlines the importance of monitoring effectiveness over time. There is therefore a need to strengthen pharmacovigilance within the national program for reporting efficacy and adverse drug reactions of various VL treatment regimens under the national road map in endemic regions.

The limitations of the original study have been described elsewhere [5]. The limitations of the extended follow-up were mainly related to the fact that this additional assessment was not planned in the original study [5]. Rather, the recommendation from the Indian national program to further substantiate treatment outcomes based on long-term risk of relapse for the new VL therapies came after a substantial number of patients had already completed 12 months post-treatment. The long recruitment period of the original study and the limited time available to reach the patients, resulted in a low tracing yield at extended follow up, and the wide variation of loss to follow-up between arms, making the results of this study difficult to interpret.

The late relapses found in this extensive cohort, including combination treatments, suggest that there are still deficiencies in the currently available treatment regimens for VL. There is an urgent need to improve monitoring and early signal detection of mechanisms of resistance development for existing treatments, while a sustained focus on developing new chemical entities for visceral leishmaniasis is critical.

## Supporting information

S1 Table Characteristics of patients with and without 6m and 12m follow-up data. (DOCX)

S2 Table Proportion of relapse between 0–6 months, 6–12 months and > 12 months. (DOCX)
