# Supplementary material for: Field effectiveness of new visceral leishmaniasis regimens after 1 year following treatment within public health facilities in Bihar, India
Source: PLoS Negl Trop Dis. 2019 Sep 26;13(9):e0007726. doi: 10.1371/journal.pntd.0007726 (PMC6782108; doi:10.1371/journal.pntd.0007726)
Supplement: S1 Table — (DOCX) [file pntd.0007726.s001.docx]

**S1 Table: Characteristics of patients with and without 6m and 12m follow-up data**

|  | **With 12 month outcome data N=1353*** | **Lost to follow-up at 12 months N=380** | **p value** |
| --- | --- | --- | --- |
| **Drug regimen N (col %)** |  |  |  |
| Ambisome | 710 (52.5) | 174 (45.8) | <.0001 |
| Amb+MF | 294 (21.7) | 60 (15.8) |  |
| MF+PM | 349 (25.8) | 146 (38.4) |  |
|  |  |  |  |
| **Demographic characteristics** |  |  |  |
|  |  |  |  |
| Age < 12 years N (col %) | 434 (32.1) | 91 (24.0) | 0.002 |
| Age > 12 years N (col%) | 919 (67.9) | 289 (76.0) |  |
|  |  |  |  |
| Male N (col %) | 824 (60.9) | 228 (60.0) | 0.75 |
| Female N (col %) | 529 (39.1) | 152 (40.0) |  |
|  |  |  |  |
| **Clinical characteristics** |  |  |  |
| Weeks of illness |  |  |  |
| <= 8 weeks | 1074 (79.4) | 289 (76.0) | 0.16 |
| > 8 weeks | 279 (20.6) | 91 (24.0) |  |
|  |  |  |  |
| Severe anemia: No | 880 (65.0) | 278 (73.2) | 0.003 |
| Severe anemia Yes | 473 (35.0) | 102 (26.8) |  |
|  |  |  |  |
| Creatinine < =1.5 | 1320 (97,6) | 370 (97.4) | 0.83 |
| Creatinine >1.5 | 33 (2.4) | 10 (2.6) |  |
|  |  |  |  |
| Alanine aminotransferase |  |  |  |
| Normal (<200) | 1299 (96.0) | 374 (98.4) | .0230 |
| Marked elevation (>=200) N(%) | 54 (4.0) | 6 (1.6) |  |
|  |  |  |  |
| Aspartate aminotransferase |  |  |  |
| Normal (<200) | 1215 (89.8) | 353 (92.9) | 0.069 |
| Marked elevation (>=200) N(%) | 138 (10.2) | 27 (7.1) |  |

*includes 20 patients LTFU at 6 months who returned for 12 month follow-up
