# Supplementary material for: Field effectiveness of new visceral leishmaniasis regimens after 1 year following treatment within public health facilities in Bihar, India
Source: PLoS Negl Trop Dis. 2019 Sep 26;13(9):e0007726. doi: 10.1371/journal.pntd.0007726 (PMC6782108; doi:10.1371/journal.pntd.0007726)
Supplement: S2 Table — (DOCX) [file pntd.0007726.s002.docx]

**S2 Table: Proportion of Relapse between 0-6 months, 6-12 months and > 12 months**

| Time to relapse | <= 3 months  N (%) | 3 - <=6 months  N (%) | 6- <=9  months  N (%) | 9-<=12  months  N (%) | >= 12  months  N (%) | Total  N (%) | p value |
| --- | --- | --- | --- | --- | --- | --- | --- |
| **Drug regimen** |  |  |  |  |  |  |  |
| SDA | 3  (6.67) | 24  (53.3) | 14  (31.1) | 3  (6.67) | 1  (2.2) | 45  (60) | 0.0577 |
| AmB + Milt | 0  (0) | 12  (48.0) | 7  (28.0) | 2  (8.0) | 4  (26.0) | 25  33.3 |  |
| Milt + PM | 0  (0) | 0  (0) | 2  (40.0) | 2  (40.0) | 1  (20.0) | 5  6.67 |  |
| **Age < 12 years** |  |  |  |  |  |  |  |
| No | 0  (0.0) | 21  (51.22) | 13  (31.71) | 4  (9.76) | 3  (7.32) | 41  (54.67) | 0.4176 |
| Yes | 3  (8.82) | 15  (44.12) | 10  (29.41) | 3  (8.82) | 3  (8.82) | 34  (45.33) |  |
| **Gender** |  |  |  |  |  |  |  |
| Male | 2  (4.00) | 26  (52.00) | 14  (28.00) | 3  (6.00) | 5  (10.00) | 50  (66.67) | 0.4958 |
| Female | 1  (4.00) | 10  (40.00) | 9  (36.00) | 4  (16.00) | 1  (4.00) | 25  33.33 |  |
|  |  |  |  |  |  |  |  |
| Total | 3  (4.0) | 36  (48.00) | 23  (30.67) | 7  (9.33) | 6  (8.00) | 75  (100) |  |
